# Nonsurgical Endodontic Treatment of a Maxillary Central Incisor with Two Separate Roots: A Case Report

**Published:** 2010-08-15

**Authors:** Mohammad Reza Nabavizadeh, Mohammad Reza Jouyandeh, Adnan Atbaee, Mahdi Sedigh Shams

**Affiliations:** 1. Department of Endodontics, Dental School, Shiraz University of Medical Sciences, Shiraz, Iran.

**Keywords:** Incisor, Maxilla, Root Canal

## Abstract

The success of endodontic therapy requires knowledge of the internal and external dental anatomy and its variations in presentation. This case report involves endodontic treatment of a traumatized maxillary central incisor with two separate roots.

## INTRODUCTION

Elimination of infection from the root canal system and prevention of reinfection is one of the main objectives of non surgical endodontic treatment [1]. Errors in debridement and shaping and obturation of root canal system can lead to endodontic failure and therefore adequate knowledge of anatomy of the root canal system and its variations are essential. These variations can also be found in maxillary central incisors [2][3][4][5][6][7][8][9][10][11][12][13][14][15][16].

The purpose of this article is to report on the endodontic treatment of a maxillary central incisor with two distinct roots.

## CASE REPORT

A 16 years old boy was referred for endodontic treatment of his discolored maxillary left central incisor. He reported a history of athletic trauma to that tooth 8 months previously.

**Figure 1:**
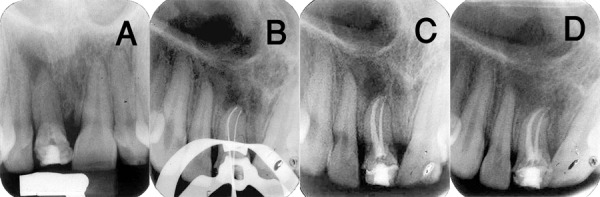
A) A pulpotomized right central incisor. Examination of the radiograph revealed the possibility of two roots. B) The working length measurement radiograph showed two files in two separated roots. C)Postoperative radiograph, two separated roots. D) Radiograph taken at six-month recall visit, showing healing of apical pathosis

Proceeding pulp vitality tests and radiographic examination, the tooth was diagnosed with asymptomatic apical periodontitis. The periapical radiograph revealed previous incomplete endodontic intervention. Moreover the tooth had a root configuration which suggested two separate roots with separate apices (Figure 1A). The periodontal ligament seemed to be widened at both apices. The tooth crown was clinically observed to be discolored when compared with the adjacent incisors.

After application of local anesthetic and rubber dam isolation, the tooth was accessed in the standard manner. Two distinct canal orifices were identified upon exploration of the pulp chamber (Figure 1B). Root canal working length was determined and then the root canal system was cleaned and shaped using Protaper Files (Dentsply, Maillefer, Ballaigues, Switzerland), Gates Glidden drills (Dentsply, Maillefer, Ballaigues, Switzerland), and sodium hypochlorite irrigant. Both canals were dried with paper points (Diadent, Tianjin, China) and obturated with gutta-percha (Orca, China) and AH-26 sealer (Denstply, Riodejaniro, RJ, Brazil), using warmed vertical condensation. The lingual access cavity was sealed with a polycarboxylate base (Hongchang Dental Equipment, China); subsequently, the tooth was restored with light cured composite resin (Shanghai A- Dental Products, China). The postoperative radiograph revealed two separate obturated canals with two separate roots (Figure 1C).

The patient was given postoperative instructions and then dismissed. At the recall examination six month post-operatively, he reported no symptoms or signs. Clinical examination also did not reveal any signs of pathology and the radiographs taken (Figure 1D) demonstrated no abnormalities and complete periapical osseous repair.

## DISCUSSION

The prevalent opinion that maxillary central incisors possess only one root with one canal can lead to endodontic treatment failure.

The present report illustrates a rare case of maxillary central incisor with two root canals, without morphological anomaly of the crown. According to literature [5], there are no limits for the morphological variability of the root canal. So practitioners must consider anatomical variation in number and architecture of root canal system. Vertucci [2] has reported that maxillary central incisor presents single root and single canal in 100% of the cases. There were few case reports describing an additional canal in the maxillary central incisor [3][4][5][6][7][8][9][10][11][12]. However, a proportion of these cases were teeth that had undergone gemination or fusion [7][11]. In these teeth canals were usually located mesiodistally to each other; however, in our study they were aligned buccolingually. Double crowns are characteristic in geminated teeth, and bifid crowns with one root is obvious in fused teeth [17]. However, in this case anomalous crown anatomy was absent. Moreover, a diagnosis of dens invagination could be disregarded due to absence of enamel and dentin invagination in the pulp on the pretreatment radiograph. Palatogingival or distolingual groove was also absent clinically and radiographically.

It may be reasonable to assume that during epithelial diaphragm formation, an incident may induce the development of a horizontal flap of hertwigs epithelial root sheet and the fusion of this horizontal flap, results in the formation of second root without any alterations in the crown [18]. However, this is a rare phenomenon and a maxillary central incisor with more than one root is an exceptional condition with only few case reports mentioned in literature [3][4][5][6][7][8][9][10][11][12].

## CONCLUSION

The experience from the present case demonstrates the variability of root canal morphology of maxillary central incisor. The clinician should be careful; even the most routine of cases might deviate from the norm.
